# Treatment Advances in Perinatal Depression: Innovations and Promising Approaches

**DOI:** 10.3390/jcm13247744

**Published:** 2024-12-18

**Authors:** Kathleen Kendall-Tackett

**Affiliations:** Texas Tech University Health Sciences Center, Amarillo, TX 79106, USA; kathleen.kendall-tackett@ttuhsc.edu

**Keywords:** pregnancy, postpartum, depression, brexaonolone, esketamine, vitamin D, infant massage, acupuncture, repetitive transcranial magnetic stimulation (rTMS)

## Abstract

**Background/Objectives**: Psychotherapy and antidepressants are the standard treatment for depression during pregnancy or postpartum. However, several new treatments for depression represent major advances and paradigm changes. This commentary highlights some innovative treatment options that are on the horizon. Most of these modalities are promising, and most are non-invasive. Many of these modalities have been used in the general population, where evidence supports their use. The methods have only recently been used for pregnant and postpartum women. Other modalities are specifically for perinatal women but do not have an established track record. **Methods**: This commentary describes some promising approaches to treatment, while acknowledging that the literature is preliminary. The goal is to highlight some interesting approaches drawn from a recent comprehensive review of the entire literature on treatment for perinatal mental illness. **Results**: Integrative treatments include vitamin D, infant massage, mindfulness-based cognitive therapy, acupuncture, and repetitive transcranial magnetic stimulation (rTMS). Many studies in the general population have found that these methods are effective, and they also show promise for perinatal women without side effects associated with medications. Some of these treatments can also be adjuncts to what is considered standard care. Two new medications, brexanolone and esketamine, quickly and effectively treat severe depression and work on GABA and glutamate receptors rather than serotonin or norepinephrine. These medications become less effective after 30 days but can be combined with selective serotonin reuptake inhibitors (SSRIs). **Conclusions**: Pregnant and postpartum women seeking care for depression and other mental health conditions have many options beyond psychotherapy and SSRI/SNRI antidepressants. These modalities can also be added to their care.

## 1. Introduction

Around the world, pregnant and postpartum women are susceptible to perinatal mental illnesses, such as depression, anxiety, and post-traumatic stress disorder (PTSD). Excessive stress, violence, trauma, and lack of support are common causes. Some groups, such as refugees, low-income women, and ethnic-minority women are at higher risk, but it can affect any woman [1]. Perinatal mental illness can negatively affect new mothers and their families. Fortunately, these conditions are treatable. If perinatal mental illness is identified and treated quickly, it can prevent years of misery [2].

Over the past two decades, psychotherapy alone, or in combination with selective serotonin reuptake inhibitors (SSRIs) or selective norepinephrine reuptake inhibitors (SNRIs), has been the standard treatment for mental illness in pregnant and postpartum women [3]. Community-based treatments, such as home visiting, can also be used to prevent or treat depression and other perinatal mental illnesses [2].

Although treatment is considered “established”, researchers have continued to explore other ways to treat perinatal depression and related conditions. These treatments are new and do not have a comprehensive evidence base—yet. In addition, some recent studies have used treatments with established efficacy in the general population and used them to treat pregnant and postpartum women.

My goal is to highlight these advances and perhaps intrigue practitioners to look further. This commentary is not intended to be a comprehensive review of the literature. Rather, I would like to draw readers’ attention to potentially promising new models of care, where evidence is emerging, but it is preliminary. This commentary is a mere taste of possibilities. I invite readers to consider these modalities as potential options.

## 2. Integrative Interventions

Medications are one important treatment option, but many new mothers will use them. Fortunately, there are other effective treatment options. Some of these treatments are new. Others have been around for centuries, but their application to perinatal women is new. Below is a summary of some promising interventions.

### 2.1. Vitamin D

Vitamin D is necessary for health and our bodies manufacture it in response to sunlight. In modern cultures, vitamin D deficiency is common because we limit sun exposure and often work indoors [4]. Although easily addressed, vitamin D deficiency is often not identified, and it increases the risk of depression because it increases systemic inflammation. Inflammation is the physiological process that underlies depression [5,6]. Ensuring that new mothers are not deficient can reduce the likelihood of depressive symptoms in new mothers because it reduces systemic inflammation.

A study of 1773 Chinese women found that 27% of pregnant and breastfeeding women were vitamin D deficient, with lower vitamin D levels correlating with higher depression scores on the Edinburgh Postnatal Depression Scale [7]. Similarly, a study from Perth, Australia, observed that women with the lowest vitamin D levels had higher depressive symptoms at 18 weeks gestation and 3 days postpartum. A systematic review of seven studies found that vitamin D deficiency was associated with increased depression risk, although most studies were observational and not randomized trials [8]. They simply compared pre-existing vitamin D levels to depression scores. While this methodology is acceptable, the evidence is not as strong as a randomized controlled trial.

Vitamin D is typically measured through serum levels of 25-hydroxyvitamin D. Severe deficiency is below 10 ng/mL, and sufficient levels are above 30 ng/mL. While the standard adult recommendation is 400 IU per day, newer guidelines suggest 4000 IU for pregnant women and 6400 IU for breastfeeding mothers, with studies indicating that these levels are safe [4,9].

### 2.2. Infant Massage

Infant massage has been an effective intervention for preterm infants for decades. Surprisingly, it also helps with maternal mental health. Human skin, and palms in particular, have oxytocin receptors. Touch can stimulate oxytocin, reducing stress and potentially lowering depression risk in mothers.

Two older trials from Britain found that infant massage significantly reduced depression symptoms in mothers, with greater improvement seen in mothers who massaged their infants compared to those who participated in support groups [10,11]. The larger of the two studies randomly assigned 34 depressed mothers to infant-massage or support-group conditions over 5 weeks [11]. Depression scores dropped for both groups, but the infant-massage group showed a larger improvement. Massage also significantly improved mother–infant interaction compared to those who received support only.

Interestingly, researchers have recently rediscovered the mental health effects of infant massage. One study recruited 138 mothers from a substance-use program [12]. Those participating in the infant-massage/parenting education group reported lower stress and depression at 12 weeks than those in control groups. The infant-massage/parenting education intervention also influenced the mothers’ physical health as reflected in their waist-to-hip ratio. Higher abdominal weight is an indication of chronic stress. The other groups did not have this sustained effect.

A study from Iran demonstrated that the effects of infant massage appear quickly in a study of 70 mothers with preterm infants [13]. Researchers assessed mothers the day before their infants were discharged from the NICU. Mothers were then randomized into either massage or usual-care conditions. Mothers in the massage group received 8 min of instruction on infant massage. On discharge day, both groups had lower anxiety, but mothers in the massage group were significantly less anxious.

### 2.3. Mindfulness-Based Cognitive Therapy (MBCT)

Cognitive therapy is a well-established psychotherapy used to treat perinatal depression, anxiety, and PTSD. MBCT is cognitive therapy that integrates mindfulness practices to break cycles of stress and negative thinking that can lead to depression. Specifically, mindfulness downregulates the stress system, which lowers the risk of depression [14]. MBCT also decreases emotional reactivity to current stressors, and it encourages present-moment awareness and non-judgmental acceptance of thoughts and feelings [15]. Mindfulness is an ancient practice recently incorporated into Western medicine. It reduces stress in adults [16]. In a meta-analysis of 41 trials involving 2993 participants, MBCT was shown to reduce anxiety, depression, and pain. It includes being aware of your breath and body and mindful movement [17].

### 2.4. Self-Compassion

Self-compassion is one aspect of mindfulness and involves treating yourself with kindness rather than judgment. It has gained attention in perinatal mental health [18]. A survey of 318 women retrospectively described their breastfeeding challenges. Mothers with more self-compassion experienced less postpartum depression and anxiety. Each increase in self-compassion decreased depressive symptoms by 41% [18].

A Chinese study adapted a Mindfulness-Based Self-Compassion Program for pregnant women with depressive symptoms [19]. Teaching self-compassion to new mothers was feasible. Women in the self-compassion group (n = 144) had less depression and parenting stress than the control group (n = 140).

### 2.5. Acupuncture

Acupuncture effectively treated major depression in pregnant women in two older American studies [20,21]. In the first study, mothers were randomly assigned to one of three conditions: acupuncture, sham acupuncture, and massage. The remission rate was 69% for acupuncture, 47% for sham acupuncture, and 32% for massage, with 20 pregnant women in each group [20]. A second study randomized 150 pregnant women with major depression into three conditions: acupuncture for depression, acupuncture that did not treat depression, and massage [21]. The remission rates were 63% for acupuncture for depression, 44% for non-specific acupuncture, and 38% for massage. There were no more recent studies on acupuncture and perinatal depression in the English literature.

However, research on acupuncture continued apace in China. One recent review of 12 randomized postpartum depression treatment trials included 443 women treated with acupuncture and 444 women in control conditions [22]. Women who received acupuncture were significantly less depressed than women in the control groups. However, the authors noted that the control groups in all the studies included active treatments such as medications, therapy, and Traditional Chinese Medicine. This may reflect differences in treatment models in China vs. the West. Western studies use acupuncture alone for a “cleaner design”, but in China, acupuncture is often combined with other modalities, including Traditional Chinese Medicine. Even with all these competing methods, acupuncture was superior to all these techniques.

Another review included 15 studies from both English and Chinese databases examining acupuncture and Chinese herbal medicine [23]. In these studies, acupuncture and Chinese herbal medicine reduced depression symptoms more effectively than a placebo or antidepressants. In fact, acupuncture was as effective as antidepressants without negative side effects. However, the authors noted the low quality of the evidence per the Cochrane risk-of-bias tool.

In Western studies, researchers have not understood why acupuncture works. One recent study used functional magnetic resonance imaging (fMRI) to map brain changes when acupuncture was used to treat postpartum depression. This study compared 52 women with postpartum depression to 24 non-depressed controls. Of the 52 women who were depressed, 22 were treated with acupuncture. Before treatment, depressed women had less gray matter volume in the amygdala than non-depressed postpartum women [24]. Acupuncture lowered depression scores, made marginal improvements in gray matter volume, and significantly enhanced resting-state values. The authors hypothesized that acupuncture decreased depression because of the structure of the amygdala and connections in brain areas that lead to negative emotions.

### 2.6. Repetitive Transcranial Magnetic Stimulation (rTMS)

rTMS is a neuromodulator that stimulates nerves. It uses a gentle magnetic pulse that stimulates the brain with tiny electrical currents that pass through magnetic coils. The patient wears a cap with these magnetic coils. It is a gentle treatment that requires no anesthesia and effectively treats major depression, obsessive–compulsive disorder (OCD), PTSD, and several diseases in the general population. European guidelines on evidence-based care found Level A (definite efficacy) or Level B (probable efficacy) for rTMS as a treatment for depression [25]. In addition, a consensus statement on rTMS, based on findings from multiple randomized trials, concluded that rTMS was a safe and effective treatment for depression [26].

Researchers are now also using it with perinatal women. A study with six women used repetitive transcranial magnetic stimulation to treat postpartum depression [27]. Depression and anxiety declined over the 4-week treatment period and depression was lower at 3 and 6 months. Of the six patients, four achieved remission. These findings are promising, but there was no control group in this study so we cannot account for the placebo effect. A review of 14 randomized trials (N = 884) from English and Chinese databases found that rTMS decreased postpartum depression and improved cognitive function [28].

One recent study provided a possible mechanism by which rTMS might decrease depressive symptoms. An fMRI study compared 32 women with postpartum depression to 32 age-matched healthy controls [29]. The depressed women had reduced connectivity in three areas of the brain (amygdala, insula, and medial frontal gyrus) compared to non-depressed women. After rTMS, connectivity in these sections is renormalized. In addition, increased insula connectivity correlated with improved depression scores. The authors concluded that postpartum depression disrupts the functional architecture of communication between hemispheres, but rTMS resets this communication.

Repetitive transcranial stimulation (rTMS) is a lesser-known treatment for depression that has a strong evidence base for its use in the general population. Studies with perinatal women have been promising. rTMS should not be confused with electroconvulsive therapy (ECT). ECT is much more severe, causes serious side effects, and requires anesthesia. In contrast, rTMS is gentle and safe, with minimal side effects, and is far preferable to ECT.

## 3. Medications for Severe Depression: Esketamine and Brexanolone

For decades, depression models have focused on monoamine neurotransmitters, such as serotonin and norepinephrine. Two large classes of antidepressants, selective serotonin reuptake inhibitors (SSRIs) and selective norepinephrine reuptake inhibitors (SNRIs), have stemmed from this research. Prozac (fluoxetine), the first SSRI, was considered a major treatment advance. This medication, and the ones that followed, were more effective, easier for primary care physicians to prescribe, and safer than tricyclic antidepressants. SSRIs still had side effects, and they took 4–6 weeks to become effective, but they were an improvement over previous antidepressants.

Esketamine and brexanolone represent another significant advance in treating depression. These medications work on an entirely different system than SSRIs/SNRIs; they affect GABA (γ-Aminobutric acid) and glutamate receptors, not serotonin or norepinephrine. Both were US Food and Drug Administration (FDA)-approved in 2019 to treat general depression (esketamine) and postpartum depression (brexonolone).

We have no trials that have used these medications for pregnant women, so caution is necessary. Prudence suggests that we do not use these medications with pregnant women unless there are no other options. Similarly, we do not how these medications affect breastfeeding women or their transfer into milk. In trials of brexanolone, breastfeeding women were specifically excluded. Caution is also warranted here as well. As these medications are meant to treat severe/suicidal depression, they would not be used for every woman with perinatal mental illness.

### 3.1. Esketamine

Esketamine (Spravato) is derived from ketamine, which is not a new medication, but has an off-label use as a treatment for severe depression and suicidal ideation [30]. Studies of ketamine found that approximately 60% of severely depressed patients treated with ketamine notice reduced symptoms within 5 h of the first dose and these effects last about 24 h [31]. Repeated dosing 2–4 times weekly sustains the results for several weeks. Ketamine is administered intravenously.

Esketamine uses only the S-isomer of ketamine and is administered intranasally, which is a significant advantage over IV infusion. Esketamine is as effective as IV ketamine, and effects occur within minutes [31]. A double-blind, randomized trial compared three different dosages of esketamine (28, 56, and 84 mg) vs. a placebo [32]. All were administered twice a week. The sample included 67 patients who had treatment-resistant major depression. Esketamine significantly improved symptoms across all dosages tested. Symptom relief was sustained even with reduced frequency of use, lasting over two months.

Because it acts so quickly, esketamine may be especially useful for patients with severe symptoms. Although used primarily for general depression, esketamine can be an option for perinatal women. It can be particularly useful to address symptoms quickly before SSRIs can be effective. Because of its potential for abuse, esketamine should only be used directly under a physician’s care.

### 3.2. Brexanolone

Approved by the FDA in 2019 specifically for postpartum depression, brexanolone also offers rapid symptom relief that lasts at least 30 days. As a synthetic form of allopregnanolone, it modulates GABA receptors with the aim of restoring brain network balance [33]. A recent review of studies of brexanolone as a treatment for postpartum depression included one small trial and two studies with larger samples that compared brexanolone to a placebo [34]. All three studies showed that brexanolone was effective for the first 30 days, and after that, there was no difference between brexanolone and SSRIs [34].

Brexanolone can also be administered via injection. A recent meta-analysis of six studies compared the efficacy of injected brexanolone with SSRIs to treat postpartum depression [35]. Initially, brexanolone was more effective than SSRIs. But by week 4 and the last observation, the gap between them diminished.

### 3.3. Zuranolone

Zuranolone, a variant of brexanolone, is orally administered once a day and does not need to be administered in a hospital. In clinical trials, zuranolone demonstrated significant symptom reduction within 3 days, with effects lasting up to 45 days [36]. A double-blind phase 3 trial randomized women with severe postpartum depression to 50 mg/day of zuranolone or a placebo for 14 days (N = 170) [33]. Women in the zuranolone group were significantly less depressed at day 15, with improvement in symptoms at days 3, 28, and 45. Another double-blind trial randomized women to 30 mg of zuranolone or a placebo for 14 days [37]. Women in the zuranolone group had lower depression and anxiety scores and improved perceived functional health compared to women in the placebo group at days 3, 15, and 45. Several authors of these studies worked for or had financial ties to the manufacturer of these medications.

## 4. Conclusions

There are many options for treating depression in pregnant and postpartum women. New fast-acting medications like brexanolone and esketamine offer relief based on targeting GABA and glutamate rather than monoamine neurotransmitters. This represents a shift in depression treatment models and is especially good news for mothers with severe depression and suicidal ideation.

But medications are not the right approach for everyone. Many non-pharmacologic treatments are also available. Two low-tech alternatives are taking vitamin D and teaching infant massage. Both are simple and can be performed at the community level. Another low-tech intervention is mindfulness and self-compassion. In this era of social media, “experts” are always available to tell mothers what they are doing wrong. Helping mothers silence these negative voices is a life skill that will last long past the postpartum period.

Acupuncture and repetitive transcranial magnetic stimulation (rTMS) represent two modalities with established track records in the general depression literature. Two advances are studies showing efficacy with perinatal depression and the number of studies from Chinese journals that are now accessible via systematic reviews. While these modalities would not be for everyone, they are innovative, gentle, effective, and safe and offer further insights into ways that treatment can modify brain activity.
